# Enhanced Drought Stress Tolerance by the Arbuscular Mycorrhizal Symbiosis in a Drought-Sensitive Maize Cultivar Is Related to a Broader and Differential Regulation of Host Plant Aquaporins than in a Drought-Tolerant Cultivar

**DOI:** 10.3389/fpls.2017.01056

**Published:** 2017-06-19

**Authors:** Gabriela Quiroga, Gorka Erice, Ricardo Aroca, François Chaumont, Juan M. Ruiz-Lozano

**Affiliations:** ^1^Departamento de Microbiología del Suelo y Sistemas Simbióticos, Estación Experimental del Zaidín – Consejo Superior de Investigaciones CientíficasGranada, Spain; ^2^Institut des Sciences de la Vie, Université catholique de LouvainLouvain-la-Neuve, Belgium

**Keywords:** aquaporins, arbuscular mycorrhizal symbiosis, drought, maize, tolerance

## Abstract

The arbuscular mycorrhizal (AM) symbiosis has been shown to improve maize tolerance to different drought stress scenarios by regulating a wide range of host plants aquaporins. The objective of this study was to highlight the differences in aquaporin regulation by comparing the effects of the AM symbiosis on root aquaporin gene expression and plant physiology in two maize cultivars with contrasting drought sensitivity. This information would help to identify key aquaporin genes involved in the enhanced drought tolerance by the AM symbiosis. Results showed that when plants were subjected to drought stress the AM symbiosis induced a higher improvement of physiological parameters in drought-sensitive plants than in drought-tolerant plants. These include efficiency of photosystem II, membrane stability, accumulation of soluble sugars and plant biomass production. Thus, drought-sensitive plants obtained higher physiological benefit from the AM symbiosis. In addition, the genes *ZmPIP1;1, ZmPIP1;3, ZmPIP1;4, ZmPIP1;6, ZmPIP2;2, ZmPIP2;4, ZmTIP1;1, and ZmTIP2;3* were down-regulated by the AM symbiosis in the drought-sensitive cultivar and only *ZmTIP4;1* was up-regulated. In contrast, in the drought-tolerant cultivar only three of the studied aquaporin genes (*ZmPIP1;6, ZmPIP2;2*, and *ZmTIP4;1*) were regulated by the AM symbiosis, resulting induced. Results in the drought-sensitive cultivar are in line with the hypothesis that down-regulation of aquaporins under water deprivation could be a way to minimize water loss, and the AM symbiosis could be helping the plant in this regulation. Indeed, during drought stress episodes, water conservation is critical for plant survival and productivity, and is achieved by an efficient uptake and stringently regulated water loss, in which aquaporins participate. Moreover, the broader and contrasting regulation of these aquaporins by the AM symbiosis in the drought-sensitive than the drought-tolerant cultivar suggests a role of these aquaporins in water homeostasis or in the transport of other solutes of physiological importance in both cultivars under drought stress conditions, which may be important for the AM-induced tolerance to drought stress.

## Introduction

Crop adaptation to new environments is of crucial importance, especially in a climate change scenario. In order to secure food production in the future, efforts need to be directed to understand the mechanisms of plant adaptation and tolerance to abiotic stresses like water shortage, as these events are expected to intensify in coming years (Elliott et al., 2014). Plants cope with drought stress by recruiting drought avoidance and/or drought tolerance mechanisms, which include osmotic adjustment, regulation of stomatal conductance and photosynthesis, production of antioxidant and scavenger compounds or regulation of water uptake and flow in their tissues (Ruiz-Lozano et al., 2012b; Candar-Cakir et al., 2016). Maize is a primary food crop, even more important than other cereals such as rice or wheat since 2012 (Min et al., 2016). The impact of drought on productivity of rice, wheat, and maize will become of capital importance, as these crops represent the 50% of total consumed calories in most populated regions (Lobell et al., 2008).

Maize is fairly susceptible to drought stress, especially in the reproductive phase, experiencing important decreases in yields under drought stress in different world regions (Daryanto et al., 2016). Indeed, maize requires more water at the later vegetative and reproductive stages that at seedlings stage, but at the early crop establishment phase, water stress also influences seedlings adaptation and their grain yield potential, because of premature flowering and a longer anthesis-silk interval (Cao and Wj, 2004; Min et al., 2016) Despite the amount of information about crop responses to water deficit, our knowledge about the mechanisms originating drought tolerance in maize seedlings is still restricted (Min et al., 2016). Previous studies of drought tolerance in maize have shown that tolerant cultivars enhanced antioxidant activity, presented lower lipid peroxidation, improved accumulation of osmolytes and turgor adjustment, maintained photosynthetic activity and regulated aquaporin genes (Anjum et al., 2016; Min et al., 2016).

In this context, the symbiosis of arbuscular mycorrhizal (AM) fungi with plant roots has been shown to be helpful to tolerate and overcome water stress episodes in different plant species (Gholamhoseini et al., 2013; Chitarra et al., 2016), including maize (Boomsma and Vyn, 2008; Bárzana et al., 2014, 2015). Authors have previously reported that AM-plant association leads to better plant antioxidant activity, osmotic regulation and root hydraulic properties (Ruiz-Lozano et al., 2012a,b). Also, AM inoculated plants generally present a higher level of photosynthetic pigments, enhanced chlorophyll fluorescence parameters and net photosynthetic rate (Yooyongwech et al., 2016), as well as, a different hormone regulation compared to control plants (Aroca et al., 2008a,b).

In maize, the improvement of physiological plant status of AM inoculated plants when subjected to drought stress has been related to a better uptake of soil nutrients and water, reduced oxidative damage, enhanced root water transport capacity, or facilitated switching between apoplastic and cell-to-cell water transport pathways (Boomsma and Vyn, 2008; Bárzana et al., 2012, 2015). Furthermore, the establishment of the AM symbiosis originates extensive morphological alterations in plant root cells, in order to accommodate the presence of an endophytic symbiont, with most of these changes concerning cytoplasmic or vacuolar membranes (Krajinski et al., 2000). Thus, it is not surprising that AM plants may present different pattern of membrane proteins such as aquaporins, candidate proteins to be involved in the exchange of nutrients and water between both organisms (Uehlein et al., 2007; Maurel and Plassard, 2011; Bárzana et al., 2014). Aquaporins are small membrane intrinsic proteins located in different cell membranes and constitute a highly diverse protein family in plants, with at least 30 isoforms in most higher plants. They transport water but some of them can also facilitate the membrane diffusion of other relevant molecules for the plant such as CO_2_, silicon, boron, urea, or ammonia (Li et al., 2014). Recently, oxygen has also been shown to be transported by several *Nicotiana tabacum* aquaporins, with NtPIP1;3 as the most promising one, which points to the significance of pore-mediated O_2_ transport for respiration and opens new perspectives for aquaporins roles in plant physiology (Zwiazek et al., 2017). Each aquaporin isoform often contributes, in concert with other isoforms, to several physiological functions. Thus, their numerous functions in plant growth and development seem to be essential but not well understood yet (Chaumont and Tyerman, 2014; Li et al., 2014; Afzal et al., 2016). However, their role in the maintenance of water homeostasis in the whole plant and in the stress responses has been well established (Chaumont and Tyerman, 2014; Afzal et al., 2016), affecting the radial water flow through the cell-to-cell pathway, which is predominant under conditions of low transpiration such as under drought stress (Steudle and Peterson, 1998). To this regard, it is also remarkable that several aquaporin genes have been found to be AM-responsive in numerous plant species (Krajinski et al., 2000; Aroca et al., 2007; Guether et al., 2009; Bárzana et al., 2014; Chitarra et al., 2016; He et al., 2016; Liu et al., 2016).

There are 36 different aquaporin isoforms in maize (Chaumont et al., 2001). In a recent study, 16 out of these 36 maize aquaporins, belonging to the four maize aquaporin subfamilies (PIPs, TIPs, NIPs, and SIPs), were found to be regulated by the AM fungus *R. irregularis* (Bárzana et al., 2014). The expression of these proteins varies according to the severity of the stress and depends on the duration of the water shortage period (Bárzana et al., 2014). Essentially, these results highlight the complex regulation of these proteins in the presence of AM symbiosis and their putative role in drought alleviation (Bárzana et al., 2014). Previous studies have provided evidences that the beneficial effects of the AM symbiosis on plant stress tolerance are generally larger in plants sensitive to the imposed stress than in tolerant ones, or under more limiting growing conditions (Subramanian et al., 1995; Subramanian and Charest, 1997; Gianinazzi et al., 2010; Bonneau et al., 2013; Yooyongwech et al., 2016). This has been emphasized also for maize plants (Boomsma and Vyn, 2008). Thus, the above approach can be combined with the use of drought-sensitive and drought-tolerant cultivars for comparative analyses (Rigano et al., 2014; Zhang et al., 2016) and for identification of key aquaporins whose expression is altered by the AM symbiosis in the sensitive cultivar to render it more tolerant (Subramanian and Charest, 1997; Yooyongwech et al., 2016). The present study deals with the hypothesis that aquaporin regulation by the AM symbiosis plays a significant role in the improvement of host plant tolerance to drought stress. Under such situation, aquaporin modulation mediated by the AM symbiosis could lead to improvements of the use of soil water and mineral resources, resulting in higher drought tolerance. The objective was to highlight the differences in aquaporin regulation by comparing the effects of AM symbiosis on root aquaporin gene expression and plant physiology in two maize cultivars with contrasting drought sensitivity. This information would help to identify key aquaporin genes involved in the enhanced drought tolerance by the AM symbiosis. A similar approach has been followed to study aquaporins involved in stomatal gating in rice plants (Vinnakota et al., 2016). Moreover, the present work deeps on the role of aquaporins in drought tolerance and their regulation by AM fungi.

## Materials and Methods

### Experimental Design and Statistical Analysis

The experiment consisted of a factorial design with two factors: (1) inoculation treatment, with non-inoculated control plants (C) and plants inoculated with the AM fungus *Rhizophagus irregularis*, strain EEZ 58 (Ri); (2) water regime, so that one half of the plants were cultivated under well-watered conditions (WW) throughout the entire experiment and the other half of the plants were subjected to drought stress for 12 days before harvest (DS). In addition, two maize cultivars with contrasting tolerance to drought stress were used. One cultivar was sensitive to drought (PR34B39) and the second was tolerant to drought (PR34G13). The different combinations of these factors gave a total of four treatments for the sensitive cultivar and four treatments for the tolerant cultivar. Ten replicates were used for each treatment, giving a total of 80 plants.

Within each maize cultivar, data were subjected to analysis of variance (ANOVA) with inoculation treatment, water regime and inoculation treatment-water regime interaction as sources of variation. *Post hoc* comparisons with the Duncan’s test were used to find out differences between groups. Within each water regime, drought-sensitive and drought-tolerant cultivars were also compared by means of Duncan’s test. The expression of the AM fungal aquaporins was analyzed by means of Student’s *T*-test.

### Soil and Biological Materials

A loamy soil was collected at the grounds of IFAPA (Granada, Spain), sieved (2 mm), diluted with quartz-sand (<1 mm) (1:1, soil:sand, v/v) and sterilized by steaming (100°C for 1 h on three consecutive days). The soil had a pH of 8.1 (water); 0.85% organic matter, nutrient concentrations (mg kg^-1^): N, 1; P, 10 (NaHCO3-extractable P); K, 110. The soil texture comprised 38.3% sand, 47.1% silt and 14.6% clay.

Maize (*Zea mays* L.) seeds from a drought-sensitive (PR34B39) and a drought-tolerant (PR34G13) cultivar were provided by Pioneer Hi-Bred, Spain (DuPont Pioneer Corporation). Seeds were pre-germinated on moist sand for 5 days and then transferred to pots filled with 1250 g of the soil/sand mixture described above.

Mycorrhizal inoculum was bulked in an open-pot culture of *Z. mays* L. and consisted of soil, spores, mycelia and infected root fragments. The AM fungus was *Rhizophagus irregularis* (Schenck and Smith), strain EEZ 58. Ten grams of inoculum with about 60 infective propagules per gram (according to the most probable number test), were added to appropriate pots at sowing time. Non-inoculated control plants received the same amount of autoclaved mycorrhizal inoculum together with a 3 ml aliquot of a filtrate (<20 μm) of the AM inoculum in order to provide a general microbial population free of AM propagules.

### Growth Conditions

The experiments were carried out under greenhouse conditions with temperatures ranging from 19 to 25°C, 16/8 light/dark period, a relative humidity of 50–60% and an average photosynthetic photon flux density of 800 μmol m^-2^ s^-1^, as measured with a light meter (LICOR, Lincoln, NE, United States, model LI-188B). Plants were cultivated for a total of 9 weeks.

Soil moisture was measured with the ML2 ThetaProbe (AT Delta-T Devices Ltd., Cambridge, United Kingdom). Water was supplied daily to maintain soil at 100% of field capacity during the first 6 weeks after sowing. The 100% soil water holding capacity corresponds to 22% volumetric soil moisture measured with the ThetaProbe, as determined experimentally in a previous experiment using a pressure plate apparatus. Then, half of the plants were allowed to dry until soil water content reached 60% of field capacity (1 day needed), while the other half were maintained at field capacity. At this stage AM and non-AM plants of both genotypes had comparable size. The 60% of soil water holding capacity corresponds to 7% volumetric soil moisture measured with the ThetaProbe (also determined experimentally with a pressure plate apparatus in a previous assay). The soil water content was daily measured with the ThetaProbe ML2 before rewatering (at the end of the afternoon), reaching a minimum soil water content around 55% of field capacity in the drought-stressed treatments. This water deficit treatment resulted in severe drought stress for maize plants, as evidenced by the decrease in stomatal conductance and efficiency of the photosystem II. The amount of water lost was added to each pot in order to keep the soil water content at the desired levels of 7% of volumetric soil moisture (Porcel and Ruiz-Lozano, 2004). Plants were maintained under such conditions for 12 additional days before harvesting.

### Measurements

#### Biomass Production and Symbiotic Development

At harvest (8 weeks after sowing) the shoot and root system of five replicates per treatment were separated and the dry weight (DW) measured after drying in a forced hot-air oven at 70°C for 2 days.

The percentage of mycorrhizal fungal colonization in maize plants was estimated by visual observation according to Phillips and Hayman (1970). The extent of mycorrhizal colonization was calculated according to the gridline intersect method (Giovannetti and Mosse, 1980) in five replicates per treatment.

#### Stomatal Conductance

Stomatal conductance was measured 2 h after the onset of photoperiod with a porometer system (Porometer AP4, Delta-T Devices Ltd., Cambridge, United Kingdom) following the user manual instructions. Stomatal conductance measurements were taken in the second youngest leaf from eight different plants of each treatment.

#### Photosynthetic Efficiency

The efficiency of photosystem II was measured with FluorPen FP100 (Photon Systems Instruments, Brno, Czechia), which allows a non-invasive assessment of plant photosynthetic performance by measuring chlorophyll a fluorescence. FluorPen quantifies the quantum yield of photosystem II as the ratio between the actual fluorescence yield in the light-adapted state (FV′) and the maximum fluorescence yield in the light-adapted state (FM′), according to Oxborough and Baker (1997). Measurements were taken in the second youngest leaf of eight different plants of each treatment.

#### Membrane Electrolyte Leakage

Leaf electrolyte leakage (EL) was determined in six plants per treatment. Leaf samples were washed with deionized water to remove surface-adhered electrolytes. The samples were placed in closed vials containing 10 mL of deionized water and incubated at 25°C on a rotary shaker (at 100 rpm) during 3 h, and the electrical conductivity of the solution (*L*_0_) was determined using a conductivity meter (Metler Toledo AG 8603, Switzerland). Samples were then placed at -80°C for 2 h. Subsequently, tubes were incubated again at room temperature under smoothly agitation and the final electrical conductivity (L_f_) was obtained after 3 h under these conditions. The EL was defined as follows: [(*L*_0_ -*L*_water_)/(*L*_f_ -*L*_water_)] × 100, where *L*_water_ is the conductivity of the deionized water used to incubate the samples.

#### Oxidative Damage to Lipids

Lipid peroxides were extracted by grinding 500 mg of fresh leaf tissues with and ice-cold mortar and 6 ml of 100 mM potassium phosphate buffer (pH 7). Homogenates were filtered through one Miracloth layer and centrifuged at 15,000 × *g* for 20 min. The chromogen was formed by mixing 200 ml of supernatants with 1 ml of a reaction mixture containing 15% (w/v) trichloroacetic acid (TCA), 0.375% (w/v) 2-thiobarbituric acid (TBA), 0.1% (w/v) butyl hydroxytoluene, 0.25 N HCl and by incubating the mixture at 100°C for 30 min (Minotti and Aust, 1987). After cooling at room temperature, tubes were centrifuged at 800 × *g* for 5 min and the supernatant was used for spectrophotometric reading at 532 nm. Lipid peroxidation was estimated as the content of 2-thiobarbituric acid-reactive substances (TBARS) and expressed as equivalents of malondialdehyde (MDA) according to Halliwell and Gutteridge (1985). The calibration curve was made using MDA in the range of 0.1–10 nmol. A blank for all samples was prepared by replacing the sample with extraction medium, and controls for each sample were prepared by replacing TBA with 0.25 N HCl. In all cases, 0.1% (w/v) butyl hydroxytoluene was included in the reaction mixtures to prevent artifactual formation of 2-TBARS during the acid-heating step of the assay.

#### Total Soluble Sugars Accumulation

At harvest, total soluble sugars (TSS) were extracted from 1 g fresh leaf tissues in 100 mM potassium phosphate buffer for TSS. Soluble sugars were analyzed by 0.025 mL of plant extract reacting with 3 ml freshly prepared anthrone [200 mg anthrone + 100 ml 72% (w:w) H_2_SO_4_] and placed in a boiling water bath for 10 min according to Irigoyen et al. (1992). After cooling, the absorbance at 620 nm was determined in a spectrophotometer Hitachi U-1900 (Hitachi Corporation, Japan). The calibration curve was made using glucose in the range of 0.2 to 0.4 mg/ml.

#### Hydrogen Peroxide Content

Hydrogen peroxide content was determined by Patterson’s method (Patterson et al., 1984; Aroca et al., 2003), with slight modifications as described previously by Aroca et al. (2003). Five hundred milligrams of fresh leaf tissues were homogenized in a cold mortar with 5 ml 5% (w/v) TCA containing 0.1 g of activated charcoal and 1% (w/v) PVPP. The homogenate was centrifuged at 18,000 × *g* for 10 min. The supernatant was filtered through a Millipore filter (0.22 mm). A volume of 1.2 ml of 100 mM potassium phosphate buffer (pH = 8.4) and 0.6 ml of the colorimetric reagent were added to 130 ml of the supernatant. The colorimetric reagent was freshly made by mixing 1:1 (v/v) 0.6 mM potassium titanium oxalate and 0.6 mM 4-2 (2-pyridylazo) resorcinol (disodium salt). The samples were incubated at 45°C for 1 h and the absorbance at 508 nm was recorded. The blanks were made by replacing leaf extract by 5% TCA.

#### Root Hydraulic Conductivity (*L*o)

Eight weeks after sowing the sap flow rate (*J*v) and *L*o were measured on detached roots exuding under atmospheric pressure for 2 h (Aroca et al., 2007). Osmotic root hydraulic conductivity (*L*o) was calculated as *L*o = *J*v/ΔΨ, where *J*v is the exuded sap flow rate and ΔΨ the osmotic potential difference between the exuded sap and the nutrient solution where the pots were immersed. These measurements were carried out 3 h after the onset of light.

#### Quantitative Real-Time RT-PCR

Total RNA was isolated from maize roots harvested at noon 8 weeks after sowing and kept at -80°C, by a phenol/chloroform extraction method followed by precipitation with LiCl (Kay et al., 1987). The RNA was subjected to DNase treatment and reverse-transcription using the QuantiTect Reverse Transcription Kit (Qiagen), following the instructions provided by manufacturer. To rule out the possibility of a genomic DNA contamination, all the cDNA sets were checked by running control PCR reactions with aliquots of the same RNA that have been subjected to the DNase treatment but not to the reverse transcription step.

The expression of the group of maize aquaporins previously selected as regulated by the AM symbiosis (Bárzana et al., 2014) was studied by real-time PCR by using iCycler system (Bio-Rad, Hercules, CA, United States) adjusting protocols to optimize the PCR reaction to each gene. The primer sets used to amplify each aquaporin gene were designed in the 3′ and 5′ untranslated regions of each gene in order to avoid unspecific amplification of the different aquaporin genes (Hachez et al., 2006; Bárzana et al., 2014). The specificity of amplicons was checked with a heat dissociation protocol (from 70 to 100°C), after the final PCR cycle. The efficiency of the primer sets was evaluated with the software Bio-Rad iQ5 (version 2.1.97.1001) by analyzing the ratio *C*t/fluorescence at four-six independent points of PCR curves (Ramakers et al., 2003), giving values between 90 and 98%. The sequences of primers used for the aquaporin and constitutive genes are those described in Bárzana et al. (2014). Standardization was carried out based on the expression of the best-performing reference gene under our growing conditions. Thus, aquaporin expression levels were normalized according to the elongation factor 1 (gi:2282583).

The fungal aquaporin genes *GintAQP1, GintAQPF1*, and *GintAQPF2* were also analyzed using the primers and conditions described previously (Aroca et al., 2009; Li et al., 2013). Standardization was carried out based on the expression of the fungal elongation factor 1a gene in each sample.

The relative abundance of transcripts was calculated by using the 2^-ΔΔct^ method (Livak and Schmittgen, 2001). Real-time PCR measurements were carried out in at least three independent RNA samples per treatment, with the threshold cycle (*C*t) determined in duplicate. Negative controls without cDNA were used in all PCR reactions.

## Results

### AM Root Colonization and Plant Biomass

**FIGURE 1:**
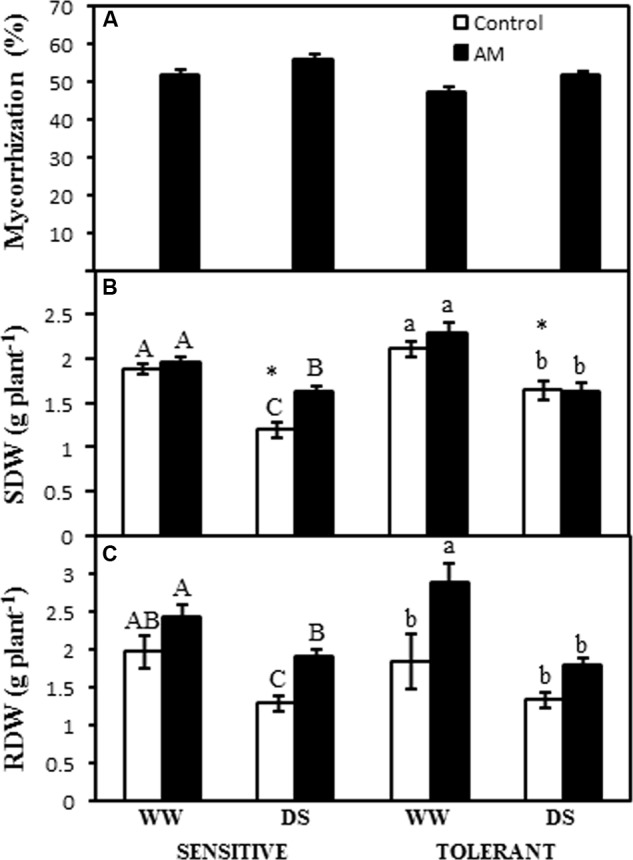
**(A)** Percentage of mycorrhizal root length, **(B)** shoot dry weight (SDW), and **(C)** root dry weight (RDW) in two maize genotypes differing in drought tolerance and inoculated or not with an arbuscular mycorrhizal (AM) fungus. Data represents the means of five values ± SE for Mycorrhization and RDW and 5 values ± SE for SDW. Different letter indicates significant differences between treatments (*p* < 0.05) based on Duncan’s test for sensitive (uppercase) and tolerant (lowercase) genotypes. Asterisks indicate significant differences between drought-sensitive and drought-tolerant genotypes within each watering regime, according to Duncan’s test.

Arbuscular mycorrhizal inoculated plants from drought-sensitive cultivar, PR34B39, had an average of 54% of mycorrhizal root length, with no significant differences due to the water treatment (**Figure 1A**). In the case of the drought-tolerant cultivar, PR34G13, mycorrhizal root length was 50%, also with no significant differences due to the water treatment. Uninoculated maize plants did not exhibit AM root colonization (**Figure 1A**).

Shoot dry weight (SDW) in the sensitive line was similar for both AM and non-AM plants when cultivated under well-watered conditions. Drought stress decreased SDW by 37% in non-AM plants but only by 17% in AM plants (**Figure 1B**). When subjected to drought stress AM plants produced 35% more SDW than non-AM plants (**Figure 1B**). In the drought-tolerant cultivar, no effect of the AM symbiosis on SDW was observed either under well-watered conditions or under drought stress. Drought stress decreased SDW by 17 and 22% in non-AM and AM plants, respectively (**Figure 1B**). In any case, under drought stress conditions, significant differences in SDW between drought-sensitive and drought-tolerant non-AM plants were observed, with the latter growing 41% more than the former (**Figure 1B**).

Drought and AM inoculation had a similar effect on root dry weight (RDW) as in SDW for both PR34B39 and PR34G13 lines (**Figure 1C**). In non-AM plants drought decreased significantly RDW in the sensitive genotype, and AM plants produced higher root biomass under drought stress conditions only in the sensitive cultivar (**Figure 1C**). In contrast, under well-watered conditions, AM plants enhanced RDW only in the tolerant cultivar.

### Stomatal Conductance (*g*s) and Efficiency of Photosystem II

**FIGURE 2:**
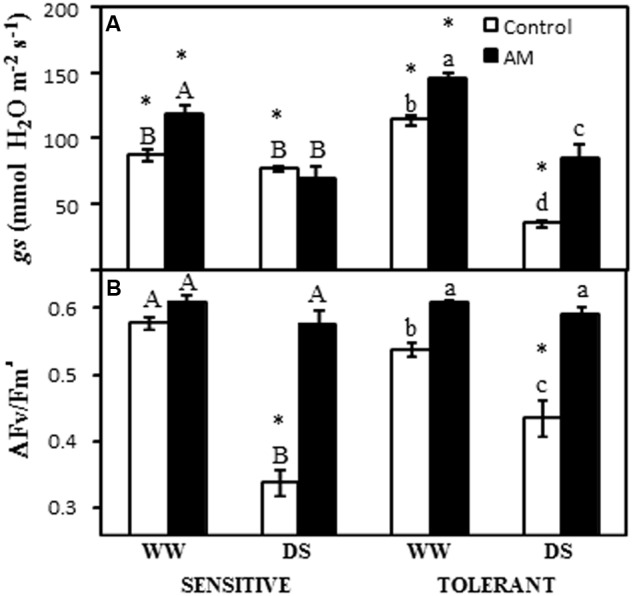
**(A)** Stomatal conductance (*g*s) and **(B)** photosystem II efficiency in the light-adapted state (Δ*F*v/*F*m′) in two maize genotypes differing in drought tolerance and inoculated or not with an AM fungus. Data represents the means of eight values ± SE. Different letter indicates significant differences between treatments (*p* < 0.05) based on Duncan’s test for sensitive (uppercase) and tolerant (lowercase) genotypes. Asterisks indicate significant differences between drought-sensitive and drought-tolerant genotypes within each watering regime, according to Duncan’s test.

The stomatal conductance (*gs*) of drought-sensitive cultivar was enhanced by the AM symbiosis under well-watered conditions (36% of increase) but not under water deficit. In the drought-sensitive cultivar drought did not significantly affect this parameter (**Figure 2A**). The drought-tolerant cultivar showed enhanced *g*s by the AM symbiosis both under well-watered conditions (27%) and under drought stress conditions (143%) (**Figure 2A**). However, drought decreased this parameter as compared to well-watered conditions. This decrease was 69% in non-AM plants and 41% in AM plants (**Figure 2A**). Under well-watered conditions both AM and non-AM plants exhibited higher *g*s values in the drought-tolerant cultivar than in the drought-sensitive one (**Figure 2A**). In contrast, under drought stress conditions, non-AM plants from the drought-tolerant cultivar had lower *g*s values than the corresponding drought-sensitive ones.

The light-adapted maximum quantum yield of PSII primary photochemistry (Δ*F*/*F*m′) in plants from drought-sensitive cultivar was affected by drought stress in the non-AM plants only, which reduced this parameter by 42% (**Figure 2B**). In contrast, in the AM plants, no significant effect was observed. In the case of the drought-tolerant cultivar, the Δ*F*/*F*m′ was enhanced by the AM symbiosis both under well-watered conditions (13% of increase) and under drought stress conditions (36% of increase) (**Figure 2B**). In this cultivar, drought stress also reduced this parameter (by 19%) in non-AM plants only (**Figure 2B**). Under drought stress conditions, significant differences in Δ*F*/*F*m′ between drought-sensitive and drought-tolerant non-AM plants were observed, with the latter having values 30% higher than the former (**Figure 2B**).

### Membrane Electrolyte Leakage

**FIGURE 3:**
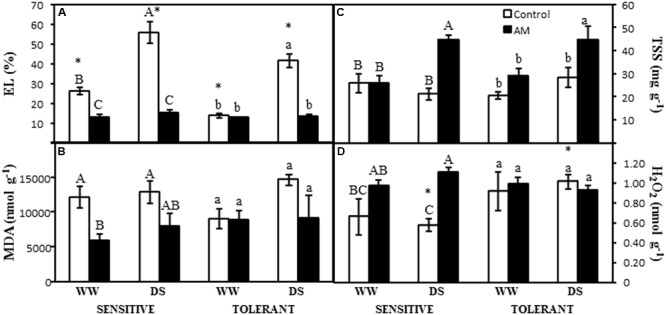
**(A)** Leaf electrolyte leakage (EL), **(B)** oxidative damage to lipids (as malondialdehyde MDA, equivalents), **(C)** total soluble sugars (TSS), and **(D)** hydrogen peroxide (H_2_O_2_) concentration in two maize genotypes differing in drought tolerance and inoculated or not with an AM fungus. Data represents the means of six values ± SE for EL and three values ± SE for MDA, TSS, and H_2_O_2_. Different letter indicates significant differences between treatments (*p* < 0.05) based on Duncan’s test for sensitive (uppercase) and tolerant (lowercase) genotypes. Asterisks indicate significant differences between drought-sensitive and drought-tolerant genotypes within each watering regime, according to Duncan’s test.

The membrane EL was reduced by the AM symbiosis in drought-sensitive plants, both under well-watered conditions (50% of decrease) and under drought stress conditions (67% of decrease) (**Figure 3A**). The imposed drought stress increased this parameter by 58% but only in non-AM plants. In the drought-tolerant cultivar the membrane EL increased by drought stress only in non-AM plants (by 279%), while AM plants did not increase this parameter as a consequence of drought (**Figure 3A**). The EL values were higher in non-AM drought-sensitive plants than in non-AM drought-tolerant ones, both under well-watered and under drought stress conditions (**Figure 3A**).

### Oxidative Damage to Lipids (MDA)

The AM symbiosis reduced the oxidative damage to lipids measured as MDA equivalents in the drought-sensitive cultivar regardless of the water regime (**Figure 3B**). In contrast, drought stress did not significantly affect this parameter either in the AM or in the non-AM plants (**Figure 3B**). In the drought-tolerant cultivar, the oxidative damage to lipids was not significantly affected by the AM symbiosis or by the drought stress imposed (**Figure 3B**).

### Total Soluble Sugars

The leaf TSS concentration was significantly increased by the AM symbiosis in both maize cultivars, but only under drought stress conditions (**Figure 3C**). Plants cultivated under well-watered conditions did not alter their TSS content as consequence of the AM symbiosis (**Figure 3C**).

### Accumulation of Hydrogen Peroxide

The accumulation of hydrogen peroxide was significantly affected by the AM symbiosis only in the drought-sensitive cultivar, increasing the values in AM plants under drought stress conditions (**Figure 3D**). Under drought stress conditions, hydrogen peroxide accumulation was higher in non-AM drought-tolerant plants than in non-AM drought-sensitive ones (**Figure 3D**).

### Root Hydraulic Conductivity (*L*o)

**FIGURE 4:**
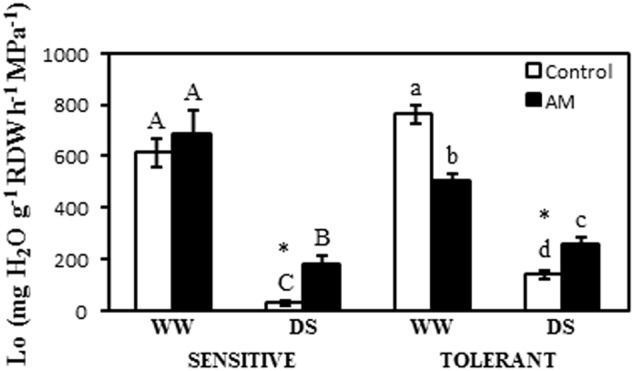
Osmotic root hydraulic conductivity (*L*o) in two maize genotypes differing in drought tolerance and inoculated or not with an AM fungus. Data represents the means of four values ± SE. Different letter indicates significant differences between treatments (*p* < 0.05) based on Duncan’s test for sensitive (uppercase) and tolerant (lowercase) genotypes. Asterisks indicate significant differences between drought-sensitive and drought-tolerant genotypes within each watering regime, according to Duncan’s test.

In the drought-sensitive cultivar root hydraulic conductivity (*L*o) was strongly reduced by drought, but this reduction reached 95% in non-AM plants and 73% in AM plants (**Figure 4**). Thus, under drought stress conditions AM plants exhibited enhanced *L*o values by five-fold when compared to non-AM plants (**Figure 4**).

In the drought-tolerant cultivar, AM symbiosis reduced *L*o by 33% under well-watered conditions but increased it by 82% under drought stress conditions. In this cultivar, drought stress also reduced significantly this parameter (**Figure 4**). Thus non-AM plants decreased *L*o by 81% as consequence of drought. The decrease was by 49% in AM plants (**Figure 4**). Under drought stress conditions, *L*o values were significantly higher (by 360%) in non-AM drought-tolerant plants than in non-AM drought-sensitive ones (**Figure 4**).

### Expression of Maize and Fungal Aquaporins

**FIGURE 5:**
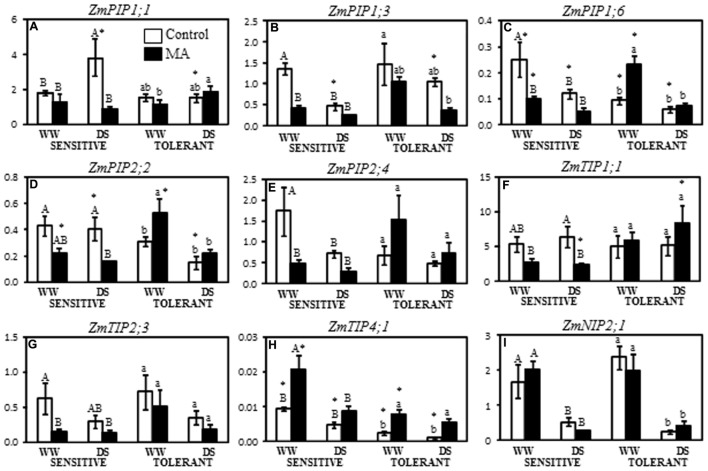
Expression of *ZmPIP1;1* **(A)**, *ZmPIP1;3* **(B)**, *ZmPIP1;6* **(C)**, *ZmPIP2;2* **(D)**, *ZmPIP2;4* **(E)**, *ZmTIP1;1* **(F)**, *ZmTIP2;3* **(G)**, *ZmTIP4;1* **(H)**, and *ZmNIP2;1* **(I)**, in two maize genotypes differing in drought tolerance and inoculated or not with an AM fungus. Values in the *Y*-axis represent the expression levels in relative units. Data represents the means of three values ± SE. Different letter indicates significant differences between treatments (*p* < 0.05) based on Duncan’s test for sensitive (uppercase) and tolerant (lowercase) genotypes. Asterisks indicate significant differences between drought-sensitive and drought-tolerant genotypes within each watering regime, according to Duncan’s test.

We analyzed the expression of 16 maize aquaporins previously shown to be regulated by the AM symbiosis under drought stress conditions (Bárzana et al., 2014). One of these genes, *ZmTIP4;2*, was not detected in any of the two genotypes, likely because of its low expression level. Besides, *ZmNIP1;1* was only detected in the sensitive genotype, but its expression was also very low, and it was not possible to detect any modification due to mycorrhization (**Supplementary Figure S1**). When analysing the expression patterns in both maize cultivars some of these aquaporin genes were not affected by the AM symbiosis or by the drought stress in the drought-tolerant cultivar (*ZmPIP1;2, ZmPIP1;4, ZmTIP1;2, ZmNIP2;2, and ZmSIP2;1*) (**Supplementary Figure S1**). The **Figure 5** shows the expression data of the aquaporin genes that are affected by the AM symbiosis and/or drought stress in both maize cultivars or at least in the drought-sensitive cultivar.

The expression of *ZmPIP1;1* in the drought-sensitive cultivar was unaltered by the AM symbiosis under well-watered conditions. However, its expression was enhanced by drought stress by 108% in non-AM plants, while in AM plants its expression did not change as consequence of drought. Thus, under drought stress conditions, the expression of *ZmPIP1;1* gene was 77% lower in AM than in non-AM plants (**Figure 5A**). On the contrary, in the drought-tolerant cultivar drought induced *ZmPIP1;1* expression by 60% in AM plants only (**Figure 5A**). Under drought stress conditions, the expression of *ZmPIP1;1* was significantly higher in non-AM drought-sensitive plants than in non-AM drought-tolerant ones (**Figure 5A**).

In the drought-sensitive cultivar the expression of *ZmPIP1;3* gene was reduced under well-watered conditions by 70% due to mycorrhization (**Figure 5B**). In the same way, the exposition to drought stress reduced the expression of this gene by 65% in non-AM plants, reaching expression values similar to those in AM plants. AM plants showed unaltered expression levels under well-watered and drought stress conditions (**Figure 5B**). In the drought-tolerant cultivar AM and non-AM plants showed no significant differences in *ZmPIP1;3* expression levels under well-watered and under drought stress conditions. Drought stress only reduced the expression of this gene in AM plants as compared to non-AM plants under well-watered conditions (**Figure 5B**). Under drought stress conditions, significant differences in the expression of *ZmPIP1;3* gene between non-AM drought-sensitive and drought-tolerant plants were observed, being higher in the latter than in the former (**Figure 5B**).

*ZmPIP1;6* was down-regulated by the AM symbiosis under well-watered conditions in the drought-sensitive cultivar, showing 60% of inhibition as compared to non-AM plants (**Figure 5C**). Drought stress inhibited the expression of this gene in non-AM plants, while in AM plants no further inhibition was observed, as compared to well-watered conditions. In the drought-tolerant cultivar, the AM symbiosis up-regulated by 150% the expression of *ZmPIP1;6* under well-watered conditions (**Figure 5C**). However, when plants were subjected to drought stress such up-regulation was avoided, reaching similar values than non-AM plants. No changes in gene expression due to water regime were observed in non-AM plants for this gene. Non-AM plants exhibited higher expression levels of *ZmPIP1;6* gene in the drought-sensitive cultivar than in the drought-tolerant one, regardless of water regime. In contrast, under well-watered conditions, AM plants had significantly higher expression levels in the drought-tolerant cultivar than in the drought-sensitive one (**Figure 5C**).

The expression of *ZmPIP2;2* in the drought-sensitive cultivar was significantly reduced by mycorrhization only under drought stress conditions (reduction by 62%) (**Figure 5D**). Under well-watered conditions this decrease was not significant. The expression of this gene in AM plants subjected to drought was also significantly lower (by 64%) than in non-AM plants under well-watered conditions. In the case of the drought-tolerant cultivar the behavior was different since AM plants up-regulated this gene by 71% under well-watered conditions. In contrast, drought stress inhibited the expression of this gene in AM plants by 58% as compared to well-watered counterparts (**Figure 5D**). In AM plants cultivated under well-watered conditions the expression of *ZmPIP2;2* was higher in the drought-tolerant cultivar than in the drought-sensitive one. The opposite was observed in non-AM plants when cultivated under drought stress conditions (**Figure 5D**).

The mRNA level of *ZmPIP2;4* was reduced by 72% by mycorrhization in the drought sensitive cultivar when cultivated under well-watered conditions (**Figure 5E**). The expression of this gene was not further inhibited by drought stress in AM plants, while in non-AM plants it was reduced by 59%. In the drought-tolerant cultivar the expression of *ZmPIP2;4* did not show significant differences due to mycorrhization or water regime (**Figure 5E**).

In the drought-sensitive cultivar the expression of *ZmTIP1;1* gene was significantly affected by the AM symbiosis only under drought stress conditions, reducing its expression levels by 63% in AM plants as compared to non-AM ones (**Figure 5F**). Drought stress itself did not significantly affect the expression of this gene in both AM and non-AM plants. In the drought-tolerant cultivar the expression of *ZmTIP1;1* was unaltered by mycorrhization or water regime (**Figure 5F**). Under drought stress conditions, the expression of *ZmTIP1;1* was significantly higher in AM drought-tolerant plants than in AM drought-sensitive plants (**Figure 5F**).

The expression of *ZmTIP2;3* in the drought sensitive cultivar was inhibited by mycorrhization when cultivated under well-watered conditions, with a reduction of 77% (**Figure 5G**). The expression of this gene was not further inhibited by drought stress. In the drought-tolerant cultivar the expression of *ZmTIP2;3* was unaltered by mycorrhization or water regime (**Figure 5G**).

The *ZmTIP4;1* expression was up-regulated under well-watered conditions in the drought-sensitive cultivar as consequence of AM root colonization, with an increase in expression levels by 122% (**Figure 5H**). However, the drought stress reduced the expression of this gene by 58%, reaching similar expression levels than non-AM plants. In the case of the drought-tolerant cultivar, the expression of *ZmTIP4;1* gene in non-AM plants was low and it was induced by the AM symbiosis both under well-watered (by 210%) and under drought stress conditions (by 310%) (**Figure 5H**). Non-AM plants had higher *ZmTIP4;1* expression levels in the drought-sensitive cultivar than in the drought-tolerant one, regardless of water regime. Moreover, under well-watered conditions, AM plants also exhibited significantly higher *ZmTIP4;1* expression in the drought-sensitive cultivar than in the drought-tolerant one (**Figure 5H**).

In the drought-sensitive cultivar the expression of *ZmNIP2;1* was only affected by drought stress, which reduced its expression in both non-AM (by 68%) and AM plants (by 87%) (**Figure 5I**). In the drought-tolerant cultivar similar data were observed, with a reduction of gene expression by drought in non-AM plants (by 90%) and in AM plants (by 79%) (**Figure 5I**).

**FIGURE 6:**
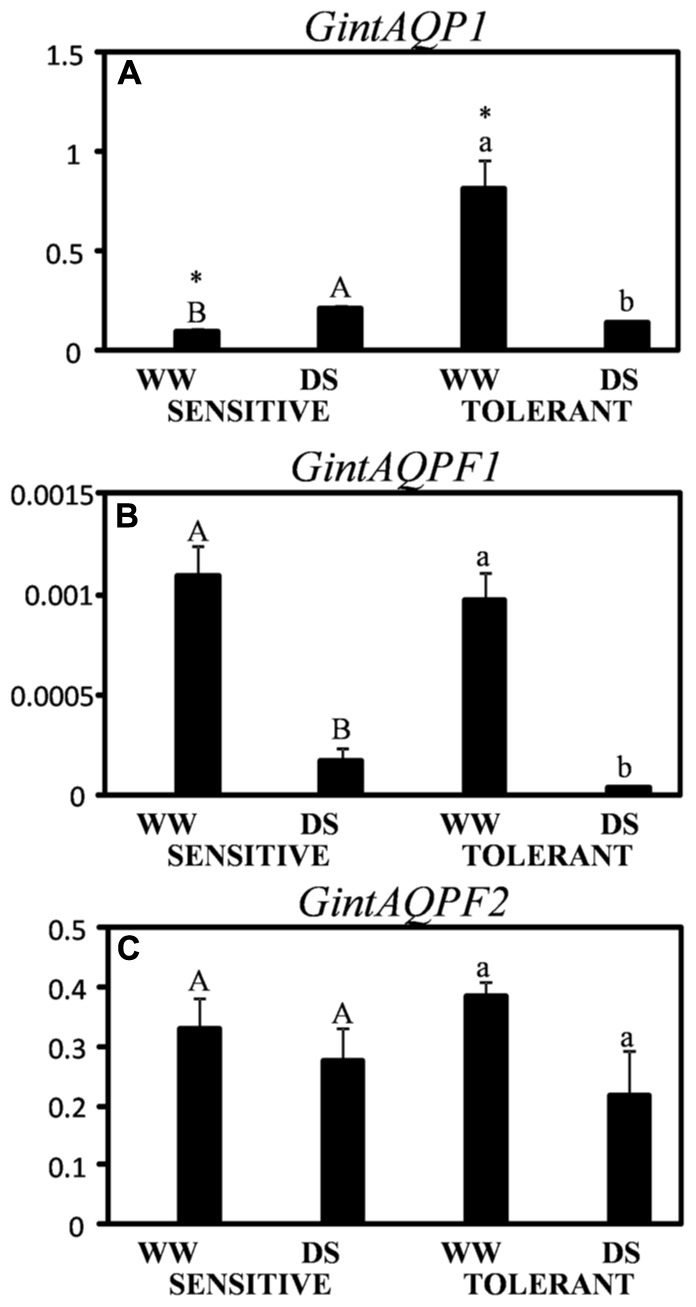
Expression of *GintAQP1* **(A)**, *GintAQPF1* **(B)**, and *GintAQPF2* **(C)** in two maize genotypes differing in drought tolerance and inoculated with the AM fungus *Rhizophagus irregularis*. Values in the *Y*-axis represent the expression levels in relative units. Data are the means of three values ± SE. Different letter indicates significant differences between treatments (*p* < 0.05) based on Student’s *T*-test for sensitive (uppercase) and tolerant (lowercase) genotypes. Asterisks indicate significant differences between drought-sensitive and drought-tolerant genotypes within each watering regime, according to Student’s *T*-test.

The expression of *GintAQP1* was slightly induced by drought stress in the drought-sensitive cultivar (**Figure 6A**). The expression of this gene was significantly higher in the drought-tolerant cultivar under well-watered conditions, but it resulted considerably inhibited (by 80%) by drought stress in this cultivar.

The gene *GintAQPF1* resulted similarly inhibited by drought stress in both cultivars (**Figure 6B**). However, the expression of this gene was lower than that of the other two fungal genes. Finally, the expression of *GintAQPF2* resulted unaltered by drought stress in both maize cultivars (**Figure 6C**).

## Discussion

This study highlights the divergent responses to AM symbiosis of two maize genotypes differing in drought tolerance: PR34G13, a drought-tolerant cultivar, and PR34B39, a drought-sensitive cultivar (DuPont Pioneer Corporation). It particularly focused on the differential regulation of root aquaporins by the AM symbiosis under well-watered and drought stress conditions and its impact on plant performance. We also featured the influence of such factors on plant growth as well as on traits showing the effects of drought and AM symbiosis on plant physiology.

### AM Effects on Plant Physiological Status

The AM fungal root colonization in both genotypes exceeded 50%, being not significantly affected by the drought stress treatment, probably due to its limited duration of only 12 days. The AM symbiosis has been previously reported to enhance drought tolerance of host plants (Augé, 2001). In the present study, the beneficial effect of the AM fungus was firstly observed in plant biomass production. Indeed, plant biomass production is an integrative index of plant performance under stressful conditions and the efficiency of the AM symbiosis has often been measured in terms of host plant biomass improvement (Ruiz-Lozano et al., 2012a). Droughted AM plants from the sensitive genotype presented higher SDW and RDW compared to non-inoculated plants. Contrariwise, no enhancement of SDW and RDW was observed in the case of the drought-tolerant genotype, highlighting genotype-depending responses to AM inoculation (Subramanian et al., 1995; Subramanian and Charest, 1997; Gianinazzi et al., 2010; Yooyongwech et al., 2016). Anyway, water deficit negatively affected growth in both maize cultivars, but to a lesser extent in the drought-tolerant genotype.

Many of the physiological responses of plants to drought stress are directed toward the control of transpiration, of root hydraulic conductivity and of osmotic adjustment (Aroca et al., 2012). Stomatal closure is a conserved mechanism in both maize genotypes studied, regardless of AM inoculation. A recent meta-analysis of 460 studies revealed that even if AM-inoculated C3 plants usually show higher *g*s values, C4 plants featured increases in *g*s of around 12% (Augé et al., 2015). In agreement with this, AM symbiosis increased *g*s in both genotypes, especially in the case of the tolerant genotype under drought conditions. However, no differences were found in *g*s values in the drought-sensitive genotype subjected to the water stress. This could be probably related to the larger SDW of these plants with the consequent increased transpiring area, or to the fact that drought-sensitive plants had generally lower *g*s values than the drought-tolerant ones. Nonetheless, it is noteworthy that the maize *g*s response to fungal inoculation could be described as inconsistent, ranging from unaltered to increased by AM (Boomsma and Vyn, 2008).

The alteration of plant physiology by AM is also confirmed with the better efficiency of Photosystem II, a highly sensitive-to-drought component from the plant photosynthetic apparatus (Ma et al., 1995). The highest effect of the AM symbiosis was shown under drought in the drought-sensitive genotype, with enhanced performance of PSII by 72% as compared to 36% enhancement in the drought-tolerant cultivar. This indicates that photochemical apparatus of droughted AM plants did not lose functionality in light conversion, that is the proportion of the light absorbed by chlorophyll associated with PSII, to reaction centers (Maxwell et al., 2000), as it was reported in other species under several stresses (Hajiboland et al., 2010; Porcel et al., 2015; Yooyongwech et al., 2016).

The percentage of membrane EL, an estimation of cell membrane stability, has been postulated as a good indicator of the tolerance to water stress (Ortiz et al., 2015). Accordingly, non-AM drought-tolerant plants had lower EL values than the corresponding drought-sensitive ones. In addition, whereas in the case of the tolerant genotype droughted AM inoculated plants showed steady state levels, in sensitive plants AM symbiosis helped to stabilize the membranes both under well-watered and under drought stress conditions. In this sense, the higher membrane stability is often related to lower MDA levels (Abid et al., 2016) accumulated as a result of lipid peroxidation. These results are in agreement with previous studies where MDA production was reduced by AM fungi (Liu et al., 2016). Furthermore, as expected, it is remarkable the similarity of results between EL and MDA concentration.

Plants need to maintain root osmotic potential below soil osmotic potential to take-up water. Previous studies have demonstrated that the AM fungi improve the plant osmotic adjustment by accumulation of different compounds (sugars, proline, free amino acids, etc.) (Bheemareddy and Lakshman, 2011; Sheng et al., 2011). This regulation by the AM symbiosis has been proposed as a mechanism allowing plants to grow under water stress (Ruiz-Lozano, 2003). In leaves of droughted plants, AM plants increased TSS in both genotypes, although to a lesser extent in the tolerant cultivar, suggesting an increased osmotic adjustment in AM plants during drought. The key effect of AM on sugar accumulation has been often reported under drought conditions (Wu et al., 2007; Zhang et al., 2014; Yooyongwech et al., 2016) as it is also shown here in maize plants from both sensitive and tolerant genotypes.

In this study, when plants were subjected to drought stress the AM symbiosis induced a higher improvement of physiological parameters in drought-sensitive plants than in drought-tolerant plants. These include efficiency of photosystem II, membrane stability, accumulation of soluble sugars and shoot and root DWs. Thus, drought-sensitive plants obtained greater physiological benefit from the AM symbiosis.

### AM Regulation of Root Hydraulic Properties

Osmotic root hydraulic conductivity (*L*o) can be considered as an estimation of water flow via the cell-to-cell pathway, and is highly related to the activity or density of water channels in the plasma membrane (Tyerman et al., 1999). A reduction in *L*o is usually reported in plants exposed to water deprivation (Javot and Maurel, 2002; Aroca et al., 2012) probably as a mechanism for preventing water loss. This fact is consistent with our results, as a sharp drop in root hydraulic conductivity was observed in both genotypes when submitted to water stress. However, under drought stress the drought-tolerant genotype maintained a higher *L*o values by 360% as compared to drought-sensitive genotype. Interestingly, AM increased *L*o under drought compared to control plants in both genotypes, and this enhancement is in accordance with previous studies on AM plants under drought (Porcel et al., 2005; Bárzana et al., 2014; Sánchez-Romera et al., 2016). The increase of *L*o in AM plants could be related to an increased expression of plant or fungal aquaporins (Sánchez-Romera et al., 2016). However, fungal aquaporins seem not to be involved in such increase since one gene was unaltered by drought, another gene was inhibited considerably in both maize cultivars, and the third one was only slightly induced in the drought-sensitive cultivar, but inhibited in the drought-tolerant one. The lack of a clear correlation between Lo and aquaporin gene expression (**Supplementary Table S1**) suggest that, the increase of *L*o in AM plants may be due to additional mechanisms such as increased abundance and/or activity of the plants aquaporins due to post-translational modifications of these proteins (Chaumont and Tyerman, 2014) or to changes in density or size of plasmodesmata in AM roots (Blee and Anderson, 1998). Indeed, symplastic movement of water via plasmodesmata may also contribute significantly to *L*o values (Galmés et al., 2007).

Aquaporin abundance in root cortex cells may alter *L*o, especially during water shortage (Maurel et al., 2015), where aquaporins are thought to be regulated for the maintenance of the adequate water balance (Jang et al., 2007a,b). Among them, PIPs were proved to contribute to the adaptation of plants to drought episodes, also contributing to rehydration of the whole plant after water shortage (Maurel et al., 2002). In addition to that, transcriptome analysis of drought tolerant and sensitive RILs in maize suggested that down-regulation of aquaporins is a mechanism contributing to the drought tolerance by upholding tissue turgor over longer time than drought-sensitive line (Min et al., 2016).

In the present study, 16 maize aquaporins previously shown to be regulated by the AM symbiosis under different drought scenarios (Bárzana et al., 2014) were analyzed to check a possible differential regulation by the AM symbiosis in two maize cultivars with contrasting drought sensitivity. We first observed that there were differences in the expression of several of the studied aquaporins between the drought-sensitive and the drought-tolerant genotypes. But these differences depended on the water regime and also on the presence or absence of the AM fungus. In the sensitive genotype, a general down-regulation of aquaporins by the AM symbiosis, under drought and/or well-watered conditions (*ZmPIP1;1, ZmPIP1;3, ZmPIP1;4, ZmPIP1;6, ZmPIP2;2, ZmPIP2;4, ZmTIP1;1*, and *ZmTIP2;3*) was featured (**Figure 5** and **Supplementary Figure S1**). Similar result was also found in maize by Bárzana et al. (2014) and in other plant species (Liang et al., 2013; Chitarra et al., 2016). However, AM regulation of aquaporins in the drought-tolerant genotype was weaker, and only three aquaporins (*ZmPIP1;6, ZmPIP2;2*, and *ZmTIP4;1*) were found to be altered. It is noteworthy that these three aquaporins were even up-regulated under well-watered conditions, which is an opposite behavior than in the sensitive genotype, similar to results reported by Liu et al. (2013) or Vinnakota et al. (2016) in two rice varieties and two *Malus* species with contrasting drought sensitivity. Also, upland rice and lowland rice with different responses to drought were compared to study the role of aquaporins in drought resistance and authors found important differences in PIP aquaporin transcriptional regulation in both types of rice (Lian et al., 2006).

During drought stress episodes, water conservation is critical for plant survival and productivity, and is achieved by an efficient uptake and stringently regulated water loss, in which aquaporins participate (Vinnakota et al., 2016). Our results in the drought-sensitive cultivar are in line with the hypothesis that down-regulation of aquaporins under water deprivation could be a way to minimize water loss, and the AM symbiosis could be helping the plant in this regulation. Through down-regulation of aquaporin expression, roots from the drought-sensitive plants may be preventing drought damages by reducing water flow through cell membranes and upholding tissue turgor as a response to the soil water deficit (Liang et al., 2013; Min et al., 2016). Indeed, dehydration avoidance during drought stress is a consequence of a tight balance between stomatal movements, root water uptake capacity and water distribution along plant tissues (Aroca et al., 2012; Ionenko et al., 2012). Nevertheless, the drought-tolerant genotype may not need this adjustment as other naturally occurring mechanisms such as deeper root development, improved turgor adjustment and photosynthetic efficiency or altered hormonal levels (Min et al., 2016) protected this genotype from the damage produced by drought.

It is also remarkable that under drought stress conditions *ZmPIP1;1, ZmTIP1;1*, and *ZmPIP2;2* were downregulated by AM only in the drought-sensitive genotype. Among these three aquaporin genes, *ZmTIP1;1* is the most expressed TIP in maize (Chaumont et al., 2001) and, besides water, it has the capacity to transport different compounds (urea, ammonia, boron, H_2_O_2_) (Bárzana et al., 2014). *ZmPIP2;2* showed a high water permeability (Pf) when expressed in *Xenopus laevis* oocytes (Bárzana et al., 2014). Thus, such tight regulation makes sense with a fine control of water balance in roots. Moreover, the specific regulation of these aquaporins by the AM symbiosis in the drought-sensitive cultivar point out a putative role of these three aquaporins in the AM-induced tolerance to drought stress, being possible targets for future studies.

In this sense, it must be taken into account that plant aquaporins can transport water, but also many other physiological substrates such as urea, glycerol, boric acid, silicic acid, hydrogen peroxide or gaseous molecules such as carbon dioxide, ammonia, or oxygen (Heinen et al., 2014; Li et al., 2014; Zwiazek et al., 2017). Among the different plant aquaporin subfamilies, NIPs is a versatile group with high diversity of substrates and a broad range of subcellular localization patterns (Maurel et al., 2008). Regulation of NIP genes by the AM symbiosis has been shown in different plant species such as *Medicago truncatula* (Uehlein et al., 2007), *Lotus japonicus* (Giovannetti et al., 2012), *Zea mays* (Bárzana et al., 2014) or *Solanum lycopersicum* (Chitarra et al., 2016). MtNIP1 had putative plasma membrane localization and was induced by mycorrhization. LjNIP1 was expressed in the inner membrane system of arbuscule-containing cells and could transport water. ZmNIP1;1 was shown to transport glycerol as well as silicon, while ZmNIP2;2 could transport silicon. LeNIP3;1 was overexpressed in AM tomato plants subjected to drought stress. Altogether, their transport capacities and localizations suggest that the regulation of NIP genes by the AM symbiosis could be involved in cell turgor regulation and in the exchange of water and solutes between both symbionts (Uehlein et al., 2007; Giovannetti et al., 2012; Bárzana et al., 2014; Chitarra et al., 2016), which may be of physiological importance to cope with drought stress.

Given the diversity of substrates that can be transported by plant aquaporins, those isoforms regulated by the AM symbiosis may have a role in regulation of leaf and root hydraulics, stomatal movement, nutrient uptake and translocation along plant tissues, carbon fixation or signaling processes. In this context, regulation of aquaporins having urea or ammonium transport capacity suggests that these aquaporins could be involved in the fungus-based nitrogen nutrition of the host plants or in plant nitrogen mobilization and metabolism (Bárzana et al., 2014), as was also proposed for ectomycorrhizal fungi (Dietz et al., 2011). Indeed, in the AM symbiosis, ammonium is suggested to be the major nitrogen compound transferred to the host plant, with urea playing a role as an intermediate solute (Tian et al., 2010). Studies by Gustavsson et al. (2005) suggested that export of plant-derived glycerol may be important for symbiotic fungi. Thus, the regulation of plant aquaporins which can transport glycerol (i.e., ZmNIP1;1 and ZmTIP4;1) may be important for the AM symbiosis or for the plant–fungus interaction under drought stress conditions. Similarly, the regulation by the AM symbiosis of aquaporins with boron and/or silicon transport capacity could have structural functions in maize plants. Hydrogen peroxide is one of the most abundant reactive oxygen species continuously produced in the metabolism of aerobic organisms. As oxidant molecule, it reacts with various cellular targets causing cell damage, while at low concentration it acts as a signal molecule, controlling different essential processes in plants (Bienert et al., 2006). Thus, aquaporins with H_2_O_2_ transport capacity such as ZmTIP1;1 could play a key role in the detoxification of excess H_2_O_2_ generated under stress conditions, or in signaling events mediated by H_2_O_2_ (Bárzana et al., 2014). That means that elucidating the *in vivo* transport capacities of the aquaporins regulated by the AM symbiosis is required to understand the role of these proteins in the AM-induced drought tolerance.

## Conclusion

In summary, under water limiting conditions AM plants enhanced maize growth, especially in the case of the drought sensitive cultivar as reflected by the larger biomass (shoots and roots) accumulation. This beneficial effect of the AM symbiosis was linked to a better efficiency of PSII, to the higher membrane stability and to lower lipid peroxidation.

It is noteworthy that *ZmPIP1;1, ZmPIP1;3, ZmPIP1;4, ZmPIP1;6, ZmPIP2;2, ZmPIP2;4, ZmTIP1;1, ZmTIP2;3*, and *ZmTIP4;1* gene expression was regulated by the AM symbiosis in the drought-sensitive cultivar, while in the drought-tolerant cultivar only *ZmPIP1;6, ZmPIP2;2*, and *ZmTIP4;1* genes were regulated by the AM symbiosis. In the drought-sensitive cultivar, the genes *ZmPIP1;1, ZmPIP2;2, and ZmTIP1;1* were down-regulated by the AM symbiosis when the plants were subjected to drought stress. Moreover, in this cultivar the genes *ZmPIP1;3, ZmPIP1;4, ZmPIP1;6, ZmPIP2;4, ZmTIP2;3* were also down-regulated when the plants grew under well-watered conditions and only *ZmTIP4;1* was up-regulated. In the drought tolerant cultivar the three genes regulated by the AM symbiosis were indeed up-regulated under well-watered conditions and *ZmTIP4;1* was in addition up-regulated under drought stress. Thus, the broader and contrasting regulation of these aquaporins by the AM symbiosis in the drought-sensitive than the drought-tolerant cultivar suggests a role of these aquaporins in water homeostasis or in the transport of solutes of physiological importance in both cultivars under drought stress conditions, which may be important for the AM-induced tolerance to drought stress. Grondin et al. (2016) found recently that a differential regulation of PIP aquaporins in six rice varieties was related to the drought stress tolerance of these varieties. Further research on the *in vivo* transport capacities by these aquaporins is needed to understand the specific role of these proteins in the AM-induced drought tolerance.

## Author Contributions

GQ and GE initiated the experiment and performed all the experimental measurements. JR-L designed the experiment and analyzed all the results. GQ, GE, and JR-L wrote the manuscript. RA and FC discussed the results and commented on the manuscript, and all the authors approved the final version of the article.

## Conflict of Interest Statement

The authors declare that the research was conducted in the absence of any commercial or financial relationships that could be construed as a potential conflict of interest.

## Supplementary Material

TABLE S1 Pearson correlation coefficients between *Lo* and expression of the different maize aquaporin genes in a drought-sensitive and a drought-tolerant genotype.

FIGURE S1 Expression of *ZmPIP1;2* **(A)**, *ZmPIP1;4* **(B)**, *ZmTIP1;2* **(C)**, *ZmNIP1;1* **(D)**, *ZmNIP2;2* **(E)**, and *ZmSIP2;1* **(F)** in two maize genotypes differing in drought tolerance and inoculated or not with an AM fungus. Data represents the means of three values ± SE. Different letter indicates significant differences between treatments (*p* < 0.05) based on Duncan’s test for sensitive (uppercase) and tolerant (lowercase) genotypes. Asterisks indicate significant differences between drought-sensitive and drought-tolerant genotypes within each watering regime, according to Duncan’s test.

## References

[B1] Abid M. Tian Z. Ata-Ul-Karim S. T. Liu Y. Cui Y. Zahoor R. (2016). Improved tolerance to post-anthesis drought stress by pre-drought priming at vegetative stages in drought-tolerant and -sensitive wheat cultivars. *Plant Physiol. Biochem.* 106 218–227. 10.1016/j.plaphy.2016.05.003 27179928

[B2] Afzal Z. Howton T. Sun Y. Mukhtar M. (2016). The roles of aquaporins in plant stress responses. *J. Dev. Biol.* 4 9 10.3390/jdb4010009 PMC5831814 29615577

[B3] Anjum S. A. Tanveer M. Ashraf U. Hussain S. Shahzad B. Khan I. (2016). Effect of progressive drought stress on growth, leaf gas exchange, and antioxidant production in two maize cultivars. *Environ. Sci. Pollut. Res.* 23 17132–17141. 10.1007/s11356-016-6894-8 27215981

[B4] Aroca R. Bago A. Sutka M. Paz J. A. Cano C. Amodeo G. (2009). Expression analysis of the first arbuscular mycorrhizal fungi aquaporin described reveals concerted gene expression between salt-stressed and nonstressed mycelium. *Mol. Plant Microbe. Interact.* 22 1169–1178. 10.1094/MPMI-22-9-1169 19656051

[B5] Aroca R. Del Mar Alguacil M. Vernieri P. Ruiz-Lozano J. M. (2008a). Plant responses to drought stress and exogenous ABA application are modulated differently by mycorrhization in tomato and an ABA-deficient mutant (Sitiens). *Microb. Ecol.* 56 704–719. 10.1007/s00248-008-9390-y 18443845

[B6] Aroca R. Irigoyen J. J. Sánchez-Díaz M. (2003). Drought enhances maize chilling tolerance. II. Photosynthetic traits and protective mechanisms against oxidative stress. *Physiol. Plant.* 117 540–549. 12675744 10.1034/j.1399-3054.2003.00065.x

[B7] Aroca R. Porcel R. Ruiz-lozano J. M. (2007). How does arbuscular mycorrhizal symbiosis regulate root hydraulic properties and plasma membrane aquaporins in *Phaseolus vulgaris* under drought, cold or salinity stresses. *New Phytol.* 173 808–816. 10.1111/j.1469-8137.2006.01961.x 17286829

[B8] Aroca R. Porcel R. Ruiz-Lozano J. M. (2012). Regulation of root water uptake under abiotic stress conditions. *J. Exp. Bot.* 63 43–57. 10.1093/jxb/err266 21914658

[B9] Aroca R. Vernieri P. Ruiz-Lozano J. M. (2008b). Mycorrhizal and non-mycorrhizal *Lactuca sativa* plants exhibit contrasting responses to exogenous ABA during drought stress and recovery. *J. Exp. Bot.* 59 2029–2041. 10.1093/jxb/ern057 18469324 PMC2413270

[B10] Augé R. M. (2001). Water relations, drought and vesicular-arbuscular mycorrhizal symbiosis. *Mycorrhiza* 11 3–42. 10.1007/s005720100097

[B11] Augé R. M. Toler H. D. Saxton A. M. (2015). Arbuscular mycorrhizal symbiosis alters stomatal conductance of host plants more under drought than under amply watered conditions: a meta-analysis. *Mycorrhiza* 25 13–24. 10.1007/s00572-014-0585-4 24831020

[B12] Bárzana G. Aroca R. Bienert G. P. Chaumont F. Ruiz-Lozano J. M. (2014). New insights into the regulation of aquaporins by the arbuscular mycorrhizal symbiosis in maiz. *Mol. Plant Microbe Interact.* 27 349–363. 10.1094/MPMI-09-13-0268-R 24593244

[B13] Bárzana G. Aroca R. Paz J. A. Chaumont F. Martinez-Ballesta M. C. Carvajal M. (2012). Arbuscular mycorrhizal symbiosis increases relative apoplastic water flow in roots of the host plant under both well-watered and drought stress conditions. *Ann. Bot.* 109 1009–1017. 10.1093/aob/mcs007 22294476 PMC3310489

[B14] Bárzana G. Aroca R. Ruiz-Lozano J. M. (2015). Localized and non-localized effects of arbuscular mycorrhizal symbiosis on accumulation of osmolytes and aquaporins and on antioxidant systems in maize plants subjected to total or partial root drying. *Plant Cell Environ.* 38 1613–1627. 10.1111/pce.12507 25630435

[B15] Bheemareddy V. Lakshman H. (2011). Effect of AM fungus *Glomus fasciculatum* on Metabolite accumulation in four varieties of *Triticum aestivum* L. Under Short-term Water stress. *Vegetos* 24 41–49.

[B16] Bienert G. P. Schjoerring J. K. Jahn T. P. (2006). Membrane transport of hydrogen peroxide. *Biochim. Biophys. Acta* 1758 994–1003. 16566894 10.1016/j.bbamem.2006.02.015

[B17] Blee K. A. Anderson A. J. (1998). Regulation of arbuscule formation by carbon in the plant. *Plant J.* 16 523–530. 10.1046/j.1365-313X.1998.00315.x

[B18] Bonneau L. Huguet S. Wipf D. Pauly N. Truong H.-N. N. (2013). Combined phosphate and nitrogen limitation generates a nutrient stress transcriptome favorable for arbuscular mycorrhizal symbiosis in *Medicago truncatula*. *New Phytol.* 199 188–202. 10.1111/nph.12234 23506613

[B19] Boomsma C. R. Vyn T. J. (2008). Maize drought tolerance: potential improvements through arbuscular mycorrhizal symbiosis? *Field Crops Res.* 108 14–31. 10.1016/j.fcr.2008.03.002

[B20] Candar-Cakir B. Arican E. Zhang B. (2016). Small RNA and degradome deep sequencing reveals drought-and tissue-specific micrornas and their important roles in drought-sensitive and drought-tolerant tomato genotypes. *Plant Biotechnol. J.* 14 1727–1746. 10.1111/pbi.12533 26857916 PMC5067666

[B21] Cao L. Z. X. Wj B. X. P. (2004). Discuss on evaluating method to drought-resistance of maize in seedling stage. *J. Maize Sci.* 12 73–75.

[B22] Chaumont F. Barrieu F. Wojcik E. Chrispeels M. J. Jung R. (2001). Aquaporins constitute a large and highly divergent protein family in maize. *Plant Physiol.* 125 1206–1215. 10.1104/pp.125.3.1206 11244102 PMC65601

[B23] Chaumont F. Tyerman S. D. (2014). Aquaporins: highly regulated channels controlling plant water relations. *Plant Physiol.* 164 1600–1618. 10.1104/pp.113.233791 24449709 PMC3982727

[B24] Chitarra W. Pagliarani C. Maserti B. Lumini E. Siciliano I. Cascone P. (2016). Insights on the impact of arbuscular mycorrhizal symbiosis on tomato tolerance to water stress. *Plant Physiol.* 171 1009–1023. 10.1104/pp.16.00307 27208301 PMC4902612

[B25] Daryanto S. Wang L. Jacinthe P. A. (2016). Global synthesis of drought effects on maize and wheat production. *PLoS ONE* 11:e0156362 10.1371/journal.pone.0156362 PMC4880198 27223810

[B26] Dietz S. Von Bülow J. Beitz E. Nehls U. (2011). The aquaporin gene family of the ectomycorrhizal fungus *Laccaria bicolor*: lessons for symbiotic functions. *New Phytol.* 190 927–940. 10.1111/j.1469-8137.2011.03651.x 21352231

[B27] Elliott J. Deryng D. Müller C. Frieler K. Konzmann M. Gerten D. (2014). Constraints and potentials of future irrigation water availability on agricultural production under climate change. *Proc. Natl. Acad. Sci. U.S.A.* 111 3239–3244. 10.1073/pnas.1222474110 24344283 PMC3948288

[B28] Galmés J. Medrano H. Flexas J. (2007). Photosynthetic limitations in response to water stress and recovery in Mediterranean plants with different growth forms. *New Phytol.* 175 81–93. 10.1111/j.1469-8137.2007.02087.x 17547669

[B29] Gholamhoseini M. Ghalavand A. Dolatabadian A. Jamshidi E. Khodaei-Joghan A. (2013). Effects of arbuscular mycorrhizal inoculation on growth, yield, nutrient uptake and irrigation water productivity of sunflowers grown under drought stress. *Agric. Water Manage.* 117 106–114. 10.1016/j.agwat.2012.11.007

[B30] Gianinazzi S. Gollotte A. Binet M. N. van Tuinen D. Redecker D. Wipf D. (2010). Agroecology: the key role of arbuscular mycorrhizas in ecosystem services. *Mycorrhiza* 20 519–530. 10.1007/s00572-010-0333-3 20697748

[B31] Giovannetti M. Balestrini R. Volpe V. Guether M. Straub D. Costa A. (2012). Two putative-aquaporin genes are differentially expressed during arbuscular mycorrhizal symbiosis in *Lotus japonicus*. *BMC Plant Biol.* 12:186 10.1186/1471-2229-12-186 PMC3533510 23046713

[B32] Giovannetti M. Mosse B. (1980). An evaluation of techniques for measuring vesicular arbuscular mycorrhizal infection in roots. *New Phytol.* 84 489–500.

[B33] Grondin A. Mauleon R. Vadez V. Henry A. (2016). Root aquaporins contribute to whole plant water fluxes under drought stress in rice (*Oryza sativa* L.). *Plant Cell Environ.* 39 347–365. 10.1111/pce.12616 26226878

[B34] Guether M. Balestrini R. Hannah M. He J. Udvardi M. K. Bonfante P. (2009). Genome-wide reprogramming of regulatory networks, transport, cell wall and membrane biogenesis during arbuscular mycorrhizal symbiosis in *Lotus japonicus*. *New Phytol.* 182 200–212. 10.1111/j.1469-8137.2008.02725.x 19192192

[B35] Gustavsson S. Lebrun A.-S. Nordén K. Chaumont F. Johanson U. (2005). A novel plant major intrinsic protein in Physcomitrella patens most similar to bacterial glycerol channels. *Plant Physiol.* 139 287–295. 16113222 10.1104/pp.105.063198 PMC1203378

[B36] Hachez C. Moshelion M. Zelazny E. Cavez D. Chaumont F. (2006). Localization and quantification of plasma membrane aquaporin expression in maize primary root: a clue to understanding their role as cellular plumbers. *Plant Mol. Biol.* 62 305–323. 10.1007/s11103-006-9022-1 16845476

[B37] Hajiboland R. Aliasgharzadeh N. Laiegh S. F. Poschenrieder C. (2010). Colonization with arbuscular mycorrhizal fungi improves salinity tolerance of tomato (*Solanum lycopersicum* L.) plants. *Plant Soil* 331 313–327. 10.1007/s11104-009-0255-z

[B38] Halliwell B. Gutteridge J. M. C. (1985). The importance of free radicals and catalytic metal ions in human diseases. *Mol. Aspects Med.* 8 89–193. 10.1016/0098-2997(85)90001-9 3908871

[B39] He F. Zhang H. Tang M. (2016). Aquaporin gene expression and physiological responses of *Robinia pseudoacacia* L. to the mycorrhizal fungus *Rhizophagus irregularis* and drought stress. *Mycorrhiza* 26 311–323. 10.1007/s00572-015-0670-3 26590998

[B40] Heinen R. B. Bienert G. P. Cohen D. Chevalier A. S. Uehlein N. Hachez C. (2014). Expression and characterization of plasma membrane aquaporins in stomatal complexes of *Zea mays*. *Plant Mol. Biol.* 86 335–350. 10.1007/s11103-014-0232-7 25082269

[B41] Ionenko I. F. F. Dautova N. R. R. Anisimov A. V. V. (2012). Early changes of water diffusional transfer in maize roots under the influence of water stress. *Environ. Exp. Bot.* 76 16–23. 10.1016/j.envexpbot.2011.09.012

[B42] Irigoyen J. J. Einerich D. W. Sanchez-Diaz M. (1992). Water stress induced changes in concentrations of proline and total soluble sugars in nodulated alfalfa (*Medicago sativa*) plants. *Physiol. Plant.* 84 55–60. 10.1111/j.1399-3054.1992.tb08764.x

[B43] Jang J. Y. Lee S. H. Rhee J. Y. Chung G. C. Ahn S. J. Kang H. (2007a). Transgenic Arabidopsis and tobacco plants overexpressing an aquaporin respond differently to various abiotic stresses. *Plant Mol. Biol.* 64 621–632. 10.1007/s11103-007-9181-8 17522953

[B44] Jang J. Y. Rhee J. Y. Kim D. G. G. Chung G. C. Lee J. H. Kang H. (2007b). Ectopic expression of a foreign aquaporin disrupts the natural expression patterns of endogenous aquaporin genes and alters plant responses to different stress conditions. *Plant Cell Physiol.* 48 1331–1339. 10.1093/pcp/pcm101 17675323

[B45] Javot H. Maurel C. (2002). The role of aquaporins in root water uptake. *Ann. Bot.* 90 301–313. 10.1093/aob/mcf199 12234142 PMC4240399

[B46] Kay R. Chan A. Daly M. McPherson J. (1987). Duplication of CaMV 35S promoter sequences creates a strong enhancer for plant genes. *Science* 236 1299–1302. 17770331 10.1126/science.236.4806.1299

[B47] Krajinski F. Biela A. Schubert D. Gianinazzi-Pearson V. Kaldenhoff R. Franken P. (2000). Arbuscular mycorrhiza development regulates the mRNA abundance of *Mtaqp1* encoding a mercury-insensitive aquaporin of *Medicago truncatula*. *Planta* 211 85–90. 10.1007/s004250000263 10923707

[B48] Li G. Santoni V. Maurel C. (2014). Plant aquaporins: roles in plant physiology. *Biochim. Biophys. Acta* 1840 1574–1582. 10.1016/j.bbagen.2013.11.004 24246957

[B49] Li T. Hu Y.-J. Hao Z.-P. Li H. Wang Y.-S. Chen B.-D. (2013). First cloning and characterization of two functional aquaporin genes from an arbuscular mycorrhizal fungus *Glomus intraradices*. *New Phytol.* 197 617–630. 10.1111/nph.12011 23157494

[B50] Lian H.-L. Su W.-A. Yu X. Lane D. Sun W.-N. Tang Z.-C. (2006). Upland rice and lowland rice exhibited different PIP expression under water deficit and ABA treatment. *Cell Res.* 16 651–660. 10.1038/sj.cr.7310068 16773042

[B51] Liang W. H. Li L. Zhang F. Liu Y. X. Li M. M. Shi H. H. (2013). Effects of abiotic stress, light, phytochromes and phytohormones on the expression of *OsAQP*, a rice aquaporin gene. *Plant Growth Regul.* 69 21–27. 10.1007/s10725-012-9743-x

[B52] Liu C. Li C. Liang D. Ma F. Wang S. Wang P. (2013). Aquaporin expression in response to water-deficit stress in two *Malus* species: relationship with physiological status and drought tolerance. *Plant Growth Regul.* 70 187–197. 10.1007/s10725-013-9791-x

[B53] Liu T. Li Z. Hui C. Tang M. Zhang H. (2016). Effect of *Rhizophagus irregularis* on osmotic adjustment, antioxidation and aquaporin PIP genes expression of *Populus × canadensis* ‘Neva’ under drought stress. *Acta Physiol. Plant.* 38 191 10.1007/s11738-016-2207-6

[B54] Livak K. J. Schmittgen T. D. (2001). Analysis of relative gene expression data using real-time quantitative PCR and the 2^-ΔΔ^ ^C_T_^ Method. *Methods* 25 402–408. 10.1006/meth.2001.1262 11846609

[B55] Lobell D. B. Burke M. B. Tebaldi C. Mastrandrea M. D. Falcon W. P. Naylor R. L. (2008). Prioritizing climate change adaptation needs for food security in 2030. *Science* 319 607–610. 10.1126/science.1152339 18239122

[B56] Ma B. L. Morrison M. J. Voldeng H. D. (1995). Leaf greenness and photosynthetic rates in soybean. *Crop Sci.* 35 1411 10.2135/cropsci1995.0011183X003500050025x

[B57] Maurel C. Boursiac Y. Luu D.-T. Santoni V. Shahzad Z. Verdoucq L. (2015). Aquaporins in Plants. *Physiol. Rev.* 95 1321–1358. 10.1152/physrev.00008.2015 26336033

[B58] Maurel C. Javot H. Lauvergeat V. Gerbeau P. Tournaire C. Santoni V. (2002). Molecular physiology of aquaporins in plants. *Int. Rev. Cytol.* 215 105–148. 11952226 10.1016/s0074-7696(02)15007-8

[B59] Maurel C. Plassard C. (2011). Aquaporins: for more than water at the plant–fungus interface? *New Phytol.* 190 815–817. 10.1111/j.1469-8137.2011.03731.x 21561457

[B60] Maurel C. Verdoucq L. Luu D. T. Santoni V. (2008). Plant aquaporins: membrane channels with multiple integrated functions. *Annu. Rev. Plant Biol.* 59 595–624. 10.1146/annurev.arplant.59.032607.092734 18444909

[B61] Maxwell K. Jonhson G. N. Johnson G. (2000). Chlorophyll fluorescence - a practical guide. *J. Exp. Bot.* 51 659–668. 10.1093/jexbot/51.345.659 10938857

[B62] Min H. Chen C. Wei S. Shang X. Sun M. Xia R. (2016). Identification of drought tolerant mechanisms in maize seedlings based on transcriptome analysis of recombination inbred lines. *Front. Plant Sci.* 7:1080 10.3389/fpls.2016.01080 PMC4961006 27507977

[B63] Minotti G. Aust S. D. (1987). The requirement for iron (III) in the initiation of lipid peroxidation by iron (II) and hydrogen peroxide. *J. Biol. Chem.* 262 1098–1104. 3027077

[B64] Ortiz N. Armada E. Duque E. Roldán A. Azcón R. (2015). Contribution of arbuscular mycorrhizal fungi and/or bacteria to enhancing plant drought tolerance under natural soil conditions: effectiveness of autochthonous or allochthonous strains. *J. Plant Physiol.* 174 87–96. 10.1016/j.jplph.2014.08.019 25462971

[B65] Oxborough K. Baker N. R. (1997). Resolving chlorophyll a fluorescence images of photosynthetic efficiency into photochemical and non-photochemical components - Calculation of qP and Fv’/Fm’ without measuring Fo’. *Photosynth. Res.* 54 135–142. 10.1023/A:1005936823310

[B66] Patterson B. D. MacRae E. A. Ferguson I. B. (1984). Estimation of hydrogen peroxide in plant extracts using titanium(IV). *Anal. Biochem.* 139 487–492. 10.1016/0003-2697(84)90039-3 6476384

[B67] Phillips J. M. Hayman D. S. (1970). Improved procedures for clearing roots and staining parasitic and vesicular-arbuscular mycorrhizal fungi for rapid assessment of infection. *Trans. Br. Mycol. Soc.* 55 158–161. 10.1016/S0007-1536(70)80110-3

[B68] Porcel R. Azcón R. Ruiz-Lozano J. M. (2005). Evaluation of the role of genes encoding for dehydrin proteins (LEA D-11) during drought stress in arbuscular mycorrhizal *Glycine max* and *Lactuca sativa* plants. *J. Exp. Bot.* 56 1933–1942. 10.1093/jxb/eri188 15911559

[B69] Porcel R. Redondo-Gómez S. Mateos-Naranjo E. Aroca R. Garcia R. Ruiz-Lozano J. M. (2015). Arbuscular mycorrhizal symbiosis ameliorates the optimum quantum yield of photosystem II and reduces non-photochemical quenching in rice plants subjected to salt stress. *J. Plant Physiol.* 185 75–83. 10.1016/j.jplph.2015.07.006 26291919

[B70] Porcel R. Ruiz-Lozano J. M. (2004). Arbuscular mycorrhizal influence on leaf water potential, solute accumulation, and oxidative stress in soybean plants subjected to drought stress. *J. Exp. Bot.* 55 1743–1750. 10.1093/jxb/erh188 15208335

[B71] Ramakers C. Ruijter J. M. Lekanne Deprez R. H. Moorman A. F. M. (2003). Assumption-free analysis of quantitative real-time polymerase chain reaction (PCR) data. *Neurosci. Lett.* 339 62–66. 10.1016/S0304-3940(02)01423-4 12618301

[B72] Rigano M. M. Arena C. Di Matteo A. Sellitto S. Frusciante L. Barone A. (2014). Eco-physiological response to water stress of drought-tolerant and drought-sensitive tomato genotypes. *Plant Biosyst.* 150 1–10. 10.1080/11263504.2014.989286

[B73] Ruiz-Lozano J. M. (2003). Arbuscular mycorrhizal symbiosis and alleviation of osmotic stress. New perspectives for molecular studies. *Mycorrhiza* 13 309–317. 10.1007/s00572-003-0237-6 12690537

[B74] Ruiz-Lozano J. M. Porcel R. Azcón R. Aroca R. (2012a). Regulation by arbuscular mycorrhizae of the integrated physiological response to salinity in plants: new challenges in physiological and molecular studies. *J. Exp. Bot.* 63 695–709. 10.1093/jxb/err313 22553287

[B75] Ruiz-Lozano J. M. Porcel R. Azcón R. Bárzana G. Aroca R. (2012b). “Contribution of arbuscular mycorrhizal symbiosis to plant drought tolerance: state of the art,” in *Responses to Drought Stress: From Morphological to Molecular Features* ed. Aroca R. (Heidelberg: Springer-Verlag) 335–362.

[B76] Sánchez-Romera B. Ruiz-Lozano J. M. Zamarreño ÁM. García-Mina J. M. Aroca R. (2016). Arbuscular mycorrhizal symbiosis and methyl jasmonate avoid the inhibition of root hydraulic conductivity caused by drought. *Mycorrhiza* 26 111–122. 10.1007/s00572-015-0650-7 26070449

[B77] Sheng M. Tang M. Zhang F. Huang Y. (2011). Influence of arbuscular mycorrhiza on organic solutes in maize leaves under salt stress. *Mycorrhiza* 21 423–430. 10.1007/s00572-010-0353-z 21191619

[B78] Steudle E. Peterson C. A. (1998). How does water get through roots? *J. Exp. Bot*. 49 775–788. 10.1093/jxb/49.322.775

[B79] Subramanian K. S. Charest C. (1997). Nutritional, growth, and reproductive responses of maize (*Zea mays* L.) to arbuscular mycorrhizal inoculation during and after drought stress at tasselling. *Mycorrhiza* 7 25–32. 10.1007/s005720050159

[B80] Subramanian K. S. Charest C. Dwyer L. M. Hamilton R. I. (1995). Arbuscular mycorrhizas and water relations in maize under drought stress at tasselling. *New Phytol.* 129 643–650. 10.1111/j.1469-8137.1995.tb03033.x

[B81] Tian C. Kasiborski B. Koul R. Lammers P. J. Bucking H. Shachar-Hill Y. (2010). Regulation of the nitrogen transfer pathway in the arbuscular mycorrhizal symbiosis: gene characterization and the coordination of expression with nitrogen flux. *Plant Physiol* 153 1175–1187. 10.1104/pp.110.156430 20448102 PMC2899933

[B82] Tyerman S. D. Bohnert H. J. Maurel C. Steudle E. Smith J. A. (1999). Plant aquaporins: their molecular biology, biophysics and significance for plant water relations. *J. Exp. Bot.* 50 1055–1071. 10.1093/jexbot/50.suppl-1.1055

[B83] Uehlein N. Fileschi K. Eckert M. Bienert G. P. Bertl A. Kaldenhoff R. (2007). Arbuscular mycorrhizal symbiosis and plant aquaporin expression. *Phytochemistry* 68 122–129. 10.1016/j.phytochem.2006.09.033 17109903

[B84] Vinnakota R. Ramakrishnan A. M. Samdani A. Venugopal M. A. Ram B. S. Krishnan S. N. (2016). A comparison of aquaporin function in mediating stomatal aperture gating among drought-tolerant and sensitive varieties of rice (*Oryza sativa* L.). *Protoplasma* 253 1593–1597. 10.1007/s00709-015-0916-0 26631017

[B85] Wu Q. S. Xia R. X. Zou Y. N. Wang G. Y. (2007). Osmotic solute responses of mycorrhizal citrus (*Poncirus trifoliata*) seedlings to drought stress. *Acta Physiol. Plant.* 29 543–549. 10.1007/s11738-007-0065-y

[B86] Yooyongwech S. Samphumphuang T. Tisarum R. Theerawitaya C. Cha-um S. (2016). Arbuscular mycorrhizal fungi (AMF) improved water deficit tolerance in two different sweet potato genotypes involves osmotic adjustments via soluble sugar and free proline. *Sci. Hortic.* 198 107–117. 10.1016/j.scienta.2015.11.002

[B87] Zhang H. Ni Z. Chen Q. Guo Z. Gao W. Su X. (2016). Proteomic responses of drought-tolerant and drought-sensitive cotton varieties to drought stress. *Mol. Genet. Genomics* 291 1293–1303. 10.1007/s00438-016-1188-x 26941218

[B88] Zhang Z. Zhang J. Huang Y. (2014). Effects of arbuscular mycorrhizal fungi on the drought tolerance of *Cyclobalanopsis glauca* seedlings under greenhouse conditions. *New For.* 45 545–556. 10.1007/s11056-014-9417-9

[B89] Zwiazek J. J. Xu H. Tan X. Navarro-Ródenas A. Morte A. (2017). Significance of oxygen transport through aquaporins. *Sci. Rep.* 7:40411 10.1038/srep40411 PMC5227684 28079178

