# Supplementary material for: Enhanced Drought Stress Tolerance by the Arbuscular Mycorrhizal Symbiosis in a Drought-Sensitive Maize Cultivar Is Related to a Broader and Differential Regulation of Host Plant Aquaporins than in a Drought-Tolerant Cultivar
Source: Front Plant Sci. 2017 Jun 19;8:1056. doi: 10.3389/fpls.2017.01056 (PMC5474487; doi:10.3389/fpls.2017.01056)
Supplement: TABLE S1 — Pearson correlation coefficients between Lo and expression of the different maize aquaporin genes in a drought-sensitive and a drought-tolerant genotype. [file Table_1.DOCX]

Table 1S. Pearson correlation coefficients between *Lo* and expression of the different maize aquaporin genes in a drought-sensitive and a drought-tolerant genotype.

| **Lo Sensitive genotype** | ***PIP1;1*** | ***PIP1;2*** | ***PIP1;3*** | ***PIP1;4*** | ***PIP1;6*** | ***PIP2;2*** | ***PIP2;4*** | ***TIP1;1*** | ***TIP1;2*** | ***TIP2;3*** | ***TIP4;1*** | ***NIP1;1*** | ***NIP2;1*** | ***NIP2;2*** | ***SIP2;1*** |
| --- | --- | --- | --- | --- | --- | --- | --- | --- | --- | --- | --- | --- | --- | --- | --- |
| Pearson coef. | -0,539 | 0,474 | 0,481 | 0,53 | 0,46 | -0,03 | 0,409 | -0,3 | 0,421 | 0,285 | 0,539 | **,954^*^** | 0,874 | -0,151 | 0,512 |
| Significance | 0,461 | 0,526 | 0,519 | 0,47 | 0,54 | 0,97 | 0,591 | 0,7 | 0,579 | 0,715 | 0,461 | 0,046 | 0,126 | 0,849 | 0,488 |
| **Lo Tolerant genotype** | ***PIP1;1*** | ***PIP1;2*** | ***PIP1;3*** | ***PIP1;4*** | ***PIP1;6*** | ***PIP2;2*** | ***PIP2;4*** | ***TIP1;1*** | ***TIP1;2*** | ***TIP2;3*** | ***TIP4;1*** | ***NIP1;1*** | ***NIP2;1*** | ***NIP2;2*** | ***SIP2;1*** |
| Pearson coef. | -0,347 | -0,381 | 0,677 | 0,923 | 0,371 | 0,571 | 0,309 | -0,355 | 0,472 | 0,886 | 0,158 | nd | **,962^*^** | -0,045 | **,985^*^** |
| Significance | 0,653 | 0,619 | 0,323 | 0,077 | 0,629 | 0,429 | 0,691 | 0,645 | 0,528 | 0,114 | 0,842 | nd | 0,038 | 0,955 | 0,015 |

nd: expression non detected
