# Supplementary figures and images for: Enhanced Drought Stress Tolerance by the Arbuscular Mycorrhizal Symbiosis in a Drought-Sensitive Maize Cultivar Is Related to a Broader and Differential Regulation of Host Plant Aquaporins than in a Drought-Tolerant Cultivar
Source: Front Plant Sci. 2017 Jun 19;8:1056. doi: 10.3389/fpls.2017.01056 (PMC5474487; doi:10.3389/fpls.2017.01056)

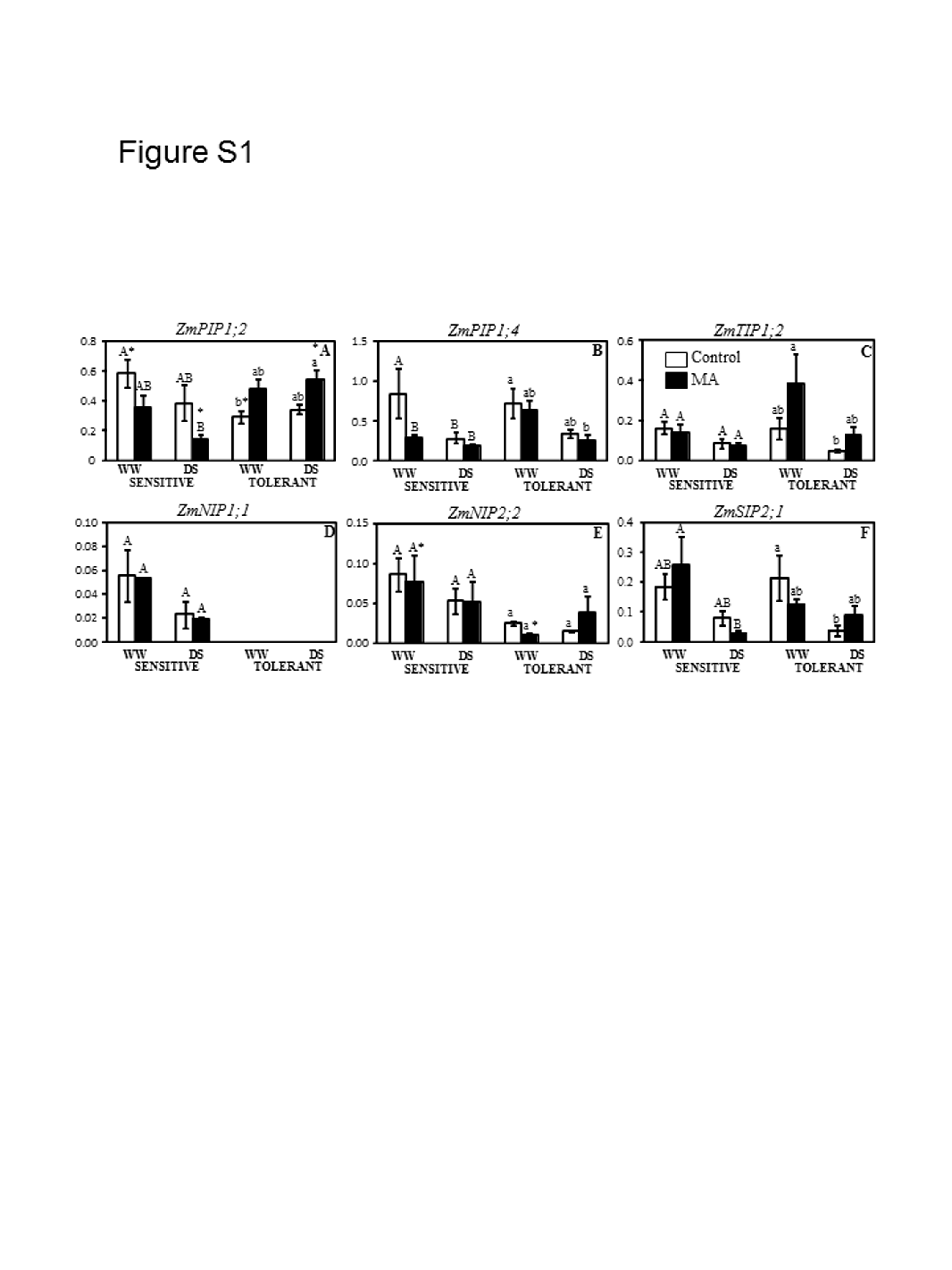

Supplement: FIGURE S1 — Expression of ZmPIP1;2 (A), ZmPIP1;4 (B), ZmTIP1;2 (C), ZmNIP1;1 (D), ZmNIP2;2 (E), and ZmSIP2;1 (F) in two maize genotypes differing in drought tolerance and inoculated or not with an AM fungus. Data represents the means of three values ± SE. Different letter indicates significant differences between treatments (p < 0.05) based on Duncan’s test for sensitive (uppercase) and tolerant (lowercase) genotypes. Asterisks indicate significant differences between drought-sensitive and drought-tolerant genotypes within each watering regime, according to Duncan’s test. [file Image_1.TIF]
